# Quantitative MRI in children with Crohn’s disease – where do we stand?

**DOI:** 10.1007/s00247-024-06033-0

**Published:** 2024-08-21

**Authors:** Pradipta Debnath, Jonathan R. Dillman

**Affiliations:** 1 Department of Radiology, Cincinnati Children’s Hospital Medical Center, 3333 Burnet Avenue, Kasota Building MLC 5031, Cincinnati, OH 45229 USA; 2 Department of Radiology, University of Cincinnati College of Medicine, Cincinnati, OH USA

**Keywords:** Child, Quantitative imaging, Crohn’s disease, Enterography, Ileum, Inflammation, Intestines, Magnetic resonance imaging

## Abstract

Crohn’s disease (CD) is a chronic inflammatory condition that affects the gastrointestinal tract, particularly the ileum and colon. This disease is characterized by recurrent bouts of intestinal inflammation with subsequent bowel wall damage, including scarring (i.e., fibrosis) and abnormal smooth muscle proliferation. MR enterography, an MRI examination tailored to assess the small bowel, is a first-line diagnostic tool for diagnosing CD in children, characterization and monitoring of disease severity and extent, and assessment of disease-related complications. To date, such MRI evaluations have been mostly qualitative, which can adversely impact diagnostic performance and inter-radiologist agreement. Quantitative MRI methods have been shown to aid in the evaluation of a variety of medical conditions and have been increasingly investigated in children and adults with CD. In CD, such objective techniques have been used to assist with diagnosis, assess treatment response, and characterize bowel wall histologic abnormalities. In the current work, we will review quantitative MRI methods for detecting and measuring intestinal active inflammation (MRI-based scoring systems, T1 relaxation mapping, diffusion-weighted imaging, intra-voxel incoherent motion, mesenteric phase contrast), bowel wall damage (magnetization transfer), and motility (quantitative cine imaging) in small bowel CD, with an emphasis on the pediatric population.

**Graphical Abstract:**

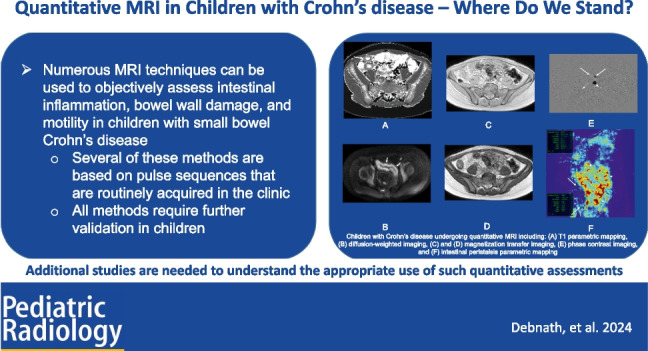

## Introduction

Crohn’s disease (CD) is a chronic inflammatory condition that can affect any portion of the gastrointestinal tract, but that most often involves the ileum and colon [1]. It is characterized by recurrent episodes of active inflammation and aberrant healing that can lead to scarring (i.e., fibrosis) and abnormal smooth muscle proliferation within the bowel wall [2]. Associated narrowing of the intestinal lumen can cause a variety of complications, including intestinal obstruction (i.e., strictures) and internal penetrating disease (e.g., fistulas and abscesses) [3].

MR enterography (MRE), an MRI examination tailored to assess the small bowel, is commonly a first-line diagnostic tool in children with CD and is used for diagnosis, characterization, and monitoring of disease severity and extent, and assessment of disease-related complications [4]. To date, such MRI evaluations have been mostly qualitative, which can adversely impact both diagnostic performance and inter-radiologist agreement [5, 6]. Quantitative MRI methods have been shown to aid in the evaluation of a variety of medical conditions and have been increasingly investigated in children and adults with CD [7]. In CD, such objective imaging techniques have been used to help with diagnosis, assessment of treatment response, and characterization of bowel wall histologic abnormalities.

Sakai et al. have recently summarized the use of quantitative MRI in small bowel Crohn’s disease with a primary focus on adults [8]. In the current work, we will review quantitative MRI methods for detecting and measuring intestinal active inflammation (MRI-based scoring systems, T1 relaxation mapping, diffusion-weighted imaging, intra-voxel incoherent motion, mesenteric phase contrast), bowel wall damage (magnetization transfer), and motility (quantitative cine imaging) in small bowel CD, with an emphasis on the pediatric population.

## Assessment of bowel wall active inflammation

Transmural active inflammation in the bowel wall is the hallmark of untreated and undertreated CD and places affected patients at increased risk for intestinal fibrosis, abnormal smooth muscle proliferation, and stricturing and internal penetrating complications. Histologically, active inflammation is characterized by the presence of an acute inflammatory infiltrate (i.e., neutrophilic inflammation with cryptitis) and mucosal ulcerations [9]. Conventional MRE findings of intestinal active inflammation include bowel wall thickening, mural edema, restricted diffusion, and postcontrast hyperenhancement as well as peri-enteric inflammation [10]. High-quality imaging also may reveal mucosal-based defects due to ulcers [10]. Numerous quantitative MRI methods have been described for evaluating bowel wall active inflammation in CD, including MRI-based scoring systems, bowel wall T1 mapping, diffusion-weighted imaging (including intra-voxel incoherent motion [IVIM]), and measurement of mesenteric blood flow.

### MRI-based scoring systems

#### Magnetic Resonance Index of Activity (MaRIA)

**Fig. 1:**
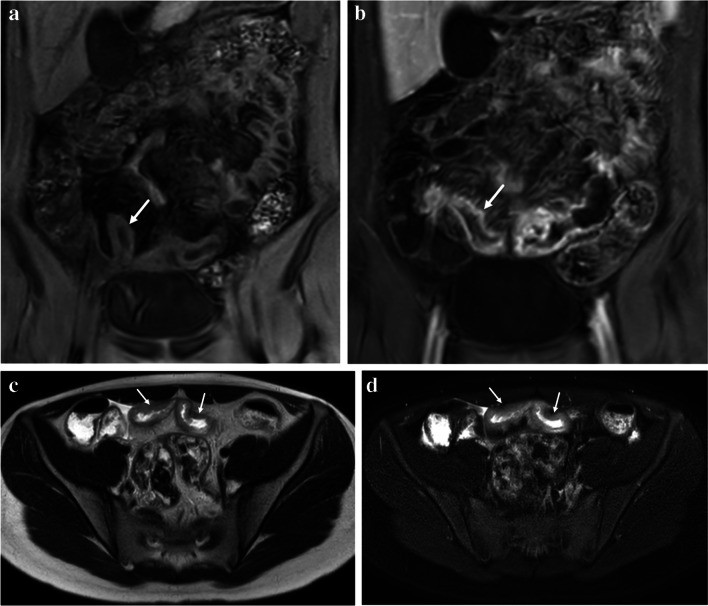
A 17-year-old female with ileal Crohn’s disease (*arrows*). **a** Coronal unenhanced T1-weighted, (**b**) coronal contrast-enhanced T1-weighted, (**c**) axial T2-weighted single-shot fast spin-echo (SSFSE), and (**d**) axial T2-weighted SSFSE with fat saturation MRI images can be used to calculate the Magnetic Resonance Index of Activity (MaRIA). Components of the MaRIA score include the degree of bowel wall thickening, presence of bowel wall edema, degree of bowel wall postcontrast hyperenhancement, and presence of radiologic ulcers (all of which are present in this patient)

The MaRIA is perhaps the most well-known MRI-based scoring system for detecting and measuring bowel wall inflammation and relies upon bowel wall thickness, relative contrast enhancement, edema, and radiologic ulcers as inputs (Fig. 1) [11]. The exact formula used to calculate this score is presented in Table 1. This scoring system, which has been mostly investigated and validated in adults and in research settings, has been shown to strongly correlate with endoscopic findings of intestinal inflammation when using the Crohn’s Disease Endoscopic Index of Severity (CDEIS) (*r*= 0.83, *P*< 0.001) [11]. A cut-off point ≥7 has been shown to predict active inflammation with a sensitivity of 0.87 and specificity of 0.87 in adults, while a cut-off point ≥11 can predict severe disease with a sensitivity of 0.92 and specificity of 0.92 [11].

**Table 1:** MRI scoring systems used to assess bowel wall active inflammation in patients with Crohn’s disease

Author	Year	Name	Formula	Positive score	Sensitivity	Specificity
Rimola [11]	2011	MaRIA	(1.5 × wall thickness) + (0.02 × relative contrast enhancement) + (5 × edema) + (10 × ulceration)	Active disease: ≥ 7	0.87^#^	0.87^#^
				Severe disease: ≥ 11	0.92^#^	0.92^#^
Ordás [14]	2019	sMaRIA	(1 × thickness > 3 mm) + (1 × edema) + (1 × fat stranding) + (2 × ulceration)	Active disease: ≥ 1	0.90^#^	0.81^#^
				Severe disease: ≥ 2	0.85^#^	0.92^#^
Focht [18]	2022	PICMI^¶^	(X^§^ × wall thickness ≥ 3 mm) + (6 × ulceration) + (9 × wall restricted diffusion) + (6 × edema) + (9 × comb sign)	Treatment response: change > 20	0.89	0.89
				“Large improvement”: change > 55	1.00	0.87
Buisson [28]	2013	Clermont score	(1.646 × wall thickness [mm])–(1.321 × ADC) + (5.613 × edema) + (8.306 × ulceration) + 5.039	Active disease: ≥ 8.4	0.83–0.85^#,$^	0.64^#,$^

*ADC*, apparent diffusion coefficient; *CI*, confidence interval; *ICC*, intraclass correlation coefficients; *MaRIA*, Magnetic Resonance Index of Activity; *PICMI*, Pediatric Inflammatory Crohn’s Magnetic Resonance Enterography Index; *sMaRIA*, Simplified Magnetic Resonance Index of Activity.

^#^Value based on adult study.

^¶^Positive score based on decrease of PICMI score between subsequent scans.

^$^Reference [29]

^§^Value of *X* changes based on wall thickness ≥ 3 mm. If wall thickness is 3 mm, then *X* = 3, 4 mm then *X* = 6, 5 mm then *X* = 9, 6 mm then *X* = 12, 7 mm then *X* = 15, etc.

In children with CD, MaRIA scores have been shown to moderately correlate with the Simplified Endoscopic Score for Crohn’s Disease (SES-CD) (*r* = 0.68, *P* < 0.005) and have good inter-reader agreement (intraclass correlation coefficient [ICC] = 0.81, *P* < 0.001) [12]. Another pediatric study also demonstrated that the MaRIA score correlated with the SES-CD (*r* = 0.70, *P* = 0.001) [13]. Similar to those in adults, primary drawbacks of the MaRIA are its need for intravenous gadolinium chelate contrast material and the complexity of the formula used to calculate the score in the clinical setting.

#### Simplified Magnetic Resonance Index of Activity (sMaRIA)

**Fig. 2:**
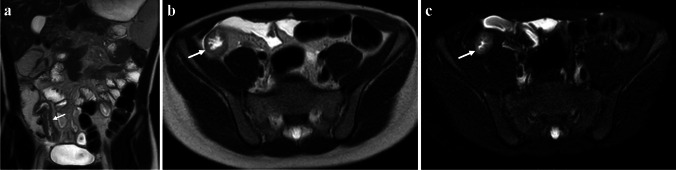
An 11-year-old male with ileal Crohn’s disease (*arrows*). **a** Coronal T2-weighted single-shot fast spin-echo (SSFSE), (**b**) axial T2-weighted SSFSE, and (**c**) axial T2-weighted SSFSE with fat saturation MR images show bowel wall thickening, intramural edema, and radiologic ulcers, yielding an simplified Magnetic Resonance Index of Activity (sMaRIA) score of 4/5. There is no peri-enteric edema

More recently, Ordás et al. developed and validated a simplified MaRIA (sMaRIA) MRI-based scoring system (Table 1) that has also been shown to correlate with both the CDEIS (*r* = 0.83, *P* < 0.001) and original MaRIA (*r* = 0.93, *P* < 0.001) [14]. This scoring system, which does not require intravenous gadolinium chelate contrast material, is semi-quantitative ranging from 0 to 5 and allocates one-point each based on the presence of bowel wall thickening (> 3 mm), bowel wall edema, and peri-enteric inflammation; two-points are allocated for the presence of radiologic ulcers (Fig. 2) [14]. In adults, an sMaRIA score ≥ 1 has been shown to accurately detect active inflammation (sensitivity of 0.90 and specificity of 0.81), and a score ≥ 2 has been shown to accurately detect severe endoscopic disease accurate (sensitivity of 0.85 and specificity of 0.92) [14].

In children with CD, substantial inter-reader agreement has been observed when using the sMaRIA (four readers: *κ* = 0.65, ICC = 0.71; *P* < 0.001 for both), and scores have been shown to correlate with the weighted pediatric Crohn’s disease activity index (wPCDAI) (*ρ* = 0.46, *P* < 0.001) and C-reactive protein (CRP) (*ρ* = 0.48; *P* < 0.001) [15]. sMaRIA scores also have been shown to significantly decrease within 6 months of starting medical therapy for CD, and they have been associated with the need for surgery in pediatric patients upon multivariable modeling [16]. Another study in children with CD concluded that the sMaRIA is accurate in reflecting disease activity in the terminal ileum but not in the colon [17].

#### Pediatric Inflammatory Crohn’s Magnetic Resonance Enterography Index (PICMI)

Focht et al. developed the Pediatric Inflammatory Crohn’s Magnetic Resonance Enterography Index (PICMI) as part of the multi-national ImageKids study. Like the sMaRIA, this MRI-based scoring system does not require intravenous gadolinium chelate contrast material [18]. Using a multivariable approach guided by experts in the field, five items were included in the final index, including wall thickness (scored if ≥ 3 mm), restricted diffusion, radiologic ulcers, mesenteric edema, and comb sign (i.e., mesenteric vascular engorgement) [18]. The exact formula used to calculate this score is presented in Table 1.

The PICMI score has been shown to correlate with the radiologist global assessment of inflammation (*r* = 0.85; *P* < 0.001) as well as the SES-CD (*r* = 0.63; *P* < 0.001) in children [18]. This scoring system also has satisfactory inter-reader agreement (ICC = 0.84, *P* < 0.001). Transmural healing, defined as PICMI ≤ 10, and therapy response, defined as a change of > 20 points, have demonstrated excellent discriminative validity (areas under the receiver operating characteristic curve = 0.92–0.97). Not surprisingly, PICMI scores highly correlate with sMaRIA scores as bowel wall thickness, radiologic ulcers, and peri-enteric inflammation are included in both indices.

### Bowel wall T1 relaxation mapping

**Fig. 3:**
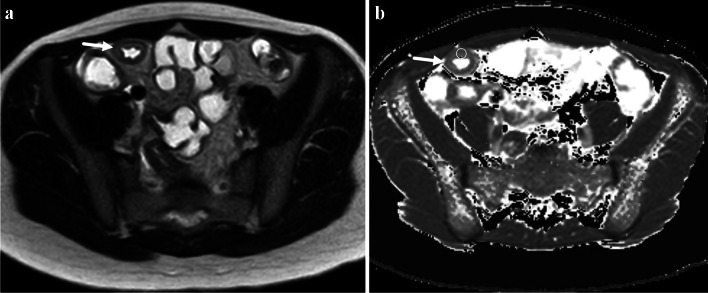
A 12-year-old female with Crohn’s disease. **a** Axial T2-weighted single-shot fast spin-echo MR image shows ileal wall thickening, intramural edema, and radiologic ulcers (*arrow*). **b** Axial T1 relaxation parametric map shows a region of interest (white circle) within the wall of the inflamed bowel (T1 = 1,361 ms) (*arrow*). Median bowel wall T1 relaxation time (ms) has been reported to be 1,159 (interquartile range 953 to 1,253) in healthy controls at 1.5-T [22]

MRI T1, or longitudinal, relaxation times are specific for a given tissue and have been shown to change with disease. These estimates have been shown to be impacted by inflammation, fibrosis, fat, edema, and iron deposition. Tissue T1 measurements have been used to detect inflammation and fibrosis in the heart and liver [19, 20]. There are few studies assessing noncontrast, or “native,” T1 relaxation estimates of the bowel wall in children or adults with CD (Fig. 3) [21, 22]. A study by Horsthuis et al. evaluated bowel wall T1 estimates, before and after intravenous gadolinium chelate injection [21].

Mahalingam et al. demonstrated that bowel wall T1 relaxation estimates are higher in children with newly diagnosed ileal CD patients (median 1,302 ms) when compared to healthy control subjects (median 1,159 ms, *P* < 0.001) [22]. These authors also showed that these measurements decrease in response to medical therapy (*P* = 0.001), and that they correlate with clinical inflammatory markers, including C-reactive protein and the weighted Pediatric Crohn’s Disease Activity Index [22]. These authors used a modified Look-Locker inversion recovery pulse sequence (5 s(3 s)3 s implementation requiring an 11-s breath-hold) with imaging performed through the most inflamed segment of terminal ileum [22].

### Bowel wall diffusion-weighted imaging (DWI)

**Fig. 4:**
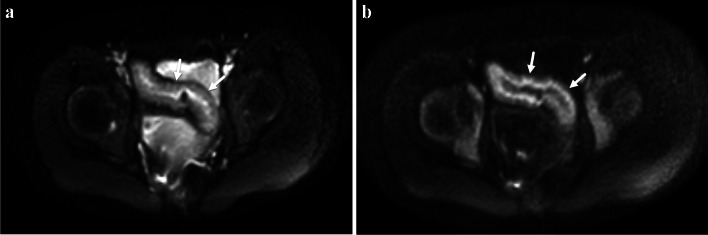
A 14-year-old male with ileal Crohn’s disease. **a** Axial low *b*-value (*b* = 0 s/mm^2^) diffusion-weighted MR image shows wall thickening of the terminal ileum (*arrows*). **b** Axial high *b*-value (*b* = 800 s/mm^2^) diffusion-weighted MR image shows restricted diffusion of water within the terminal ileum suggestive of active inflammation (*arrows*). This portion of the bowel appeared hypointense on the corresponding apparent diffusion coefficient (ADC) image

Diffusion-weighted imaging (DWI) creates a signal based on impeded diffusion of water molecules in body tissues [23]. DWI images are most often qualitatively assessed by radiologists to identify hyperintense bowel loops, an imaging feature of active inflammation (Fig. 4). Such a subjective evaluation is supported by numerous published studies [24] and is commonly included in routine clinical pediatric and adult MRE protocols [10]. The degree of impeded, or restricted, diffusion can be quantified using apparent diffusion coefficients (i.e., ADC values).

A recent meta-analysis by Kim et al. which involved nine studies that included pediatric patients with inflammatory bowel disease reported a sensitivity and specificity of DWI-MRE of 0.93 and 0.95, respectively [25]. In another pediatric cohort, Dillman et al. demonstrated that bowel wall ADC values increase over time after patients were treated with anti-tumor necrosis factor-alpha medical therapy [26]. Adding DWI to conventional MRE protocols also has been shown to improve diagnostic accuracy when evaluating children with CD [27].

A modified version of the original MaRIA score that replaces relative contrast enhancement with DWI ADC values is known as the Clermont score and does not require intravenous gadolinium chelate contrast material (Table 1) [28]. This scoring system has been shown to have good inter-observer agreement and to highly correlate with MaRIA scores when assessing the small bowel [29]. A small study in children demonstrated a moderate correlation with the SES-CD (*r* = 0.68) and good inter-reader agreement (ICC = 0.77) [12].

### Intra-voxel incoherent motion (IVIM)

**Fig. 5:**
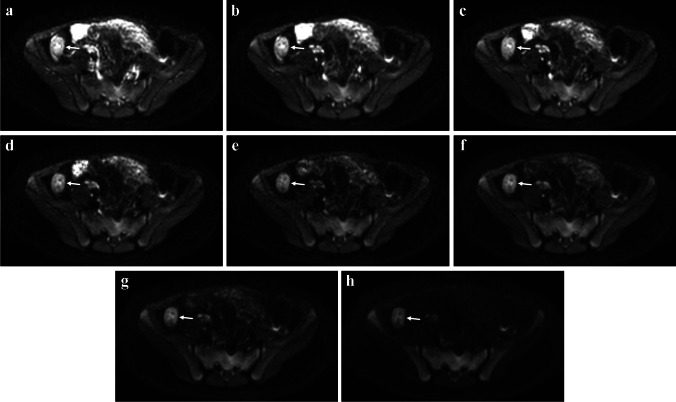
A 12-year-old female with ileal Crohn’s disease. Multiple diffusion-weighted MR images were acquired for intra-voxel incoherent motion (IVIM) assessment. **a**-**h** Normal bowel loops show progressive loss of signal intensity on eight diffusion-weighted images with increasing *b*-values (from 0 s/mm^2^ to 800 s/mm^2^). The terminal ileum appears relatively hyperintense on all eight images (*arrows*). Change in terminal ileal signal intensity vs. *b*-value was fit using a biexponential model to obtain the IVIM parameters *f* (microcirculation blood volume), D* (pseudodiffusion coefficient associated with microcirculation blood flow), and *D* (pure diffusion coefficient)

The biexponential intra-voxel incoherent motion (IVIM) approach to modeling DWI data has been proposed as an alternative to the monoexponential approach that yields ADC values. This more advanced model provides three separate quantitative parameters that reflect water diffusivity (*D*, analogous to ADC), blood flow in the microvasculature (D*), and microvascular blood volume, or perfusion fraction (*f*) from multi-*b*-value DWI data (Fig. 5) [30]. Alves et al. reported that IVIM *D* (*P* < 0.001), D* (*P* = 0.004), and *f* (*P* = 0.001) measurements were all lower in children with newly diagnosed ileal CD than in healthy control subjects [31]. The IVIM *f* (*P* = 0.016) and D* (*P* = 0.047) measurements both increased in response to medical therapy, while there was no significant change in IVIM *D* measurements (*P* = 0.10) [31]. These results suggest that more severely inflamed bowel may be relatively hypoperfused at the microvascular level (demonstrating both decreased blood volume and blood flow), a finding that has been suggested in previous pathology-based studies [32, 33].

### Measurement of mesenteric blood flow

**Fig. 6:**
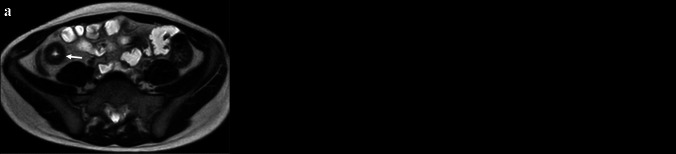
A 12-year-old male with Crohn’s disease. **a** Axial T2-weighted single-shot fast spin-echo (SSFSE) MR image shows ileal wall thickening (*arrow*). **b** Axial SSFSE through the level of the root of the small bowel mesentery shows the superior mesenteric artery and vein in cross-section (*arrows*). This anatomic level was used for phase-contrast imaging. **c** Axial velocity-encoded phase-contrast MRI image shows signal in the superior mesenteric artery and vein (*long arrows*) as well as in the abdominal aorta and inferior vena cava (*short arrows*). Blood flowing inferiorly is hypointense, while blood flowing superiorly is hyperintense

Mesenteric arterial and venous blood flow commonly increase in the setting of intestinal inflammation. This can be subjectively detected based on increased conspicuity of mesenteric vasculature adjacent to the bowel wall (i.e., the “comb sign”) [34] and bowel wall hyperenhancement on postcontrast imaging, especially early phase imaging. Abu Ata et al. objectively showed that both superior mesenteric arterial and venous blood flow are increased in the setting of small bowel CD using velocity-encoded phase-contrast MRI [35]. This technique uses bipolar gradients to encode phase shifts that are proportional to the proton velocity (Fig. 6) [36] and has been used to successfully quantify arterial and venous blood flow in other areas of the body, such as the cardiovascular system.

Abu Ata et al. hypothesized that inflamed ileum supplied and drained by ileal and ileocolic mesenteric vessels would increase both superior mesenteric arterial and venous blood flow in the root of the small bowel mesentery when normalized to abdominal aortic blood flow [35]. Their study showed that both superior mesenteric artery and superior mesenteric vein blood flow are increased in children with small bowel CD compared to healthy control subjects (*P* = 0.003 and *P* = 0.002, respectively) [35]. Furthermore, blood flow in both vessels significantly decreased by 6 weeks into medical therapy [35]. While these initial results are promising, more studies are needed to determine the clinical value of this quantitative technique for diagnosing and monitoring children and adults with small bowel CD.

## Assessment of bowel wall fibrosis

Bowel wall fibrosis occurs in patients with CD due to persistent or recurrent bouts of active inflammation with abnormal healing. This aberrant healing process is characterized by excess mural collagen and extracellular matrix that are primarily deposited in the submucosal layer of the bowel [2]. This deposition of scar tissue commonly co-exists with abnormally increased smooth muscle in the bowel wall. The literature suggests that fibrogenesis, once initiated, may continue in the absence of active inflammation [2]. Currently, there are no anti-fibrotic medications approved for use in CD in the United States. Consequently, patients with CD with substantial bowel wall fibrosis are typically managed with surgical intestinal resection, surgical stricturoplasty, or endoscopic dilation [37].

Histologic bowel wall fibrosis and active inflammation commonly co-exist. Thus, substantial bowel wall fibrosis can be present even when MRE shows findings of intestinal active inflammation, such as postcontrast hyperenhancement and restricted diffusion. Qualitative MRE findings that have been associated with the presence of bowel wall fibrosis include decreased signal intensity on T2-weighted imaging, delayed postcontrast hyperenhancement, and prestenotic dilation of the bowel > 3–4 cm [38, 39]. These features are assessed in a mostly qualitative manner when used in the clinic and are likely insensitive to early (i.e., mild and moderate) fibrosis. Most recently, the more objective stricture ratio, the ratio of maximum upstream lumen diameter to minimum lumen diameter, has also been shown to correlate with the need for bowel resection in children with stricturing ileal CD [40].

**Fig. 7:**
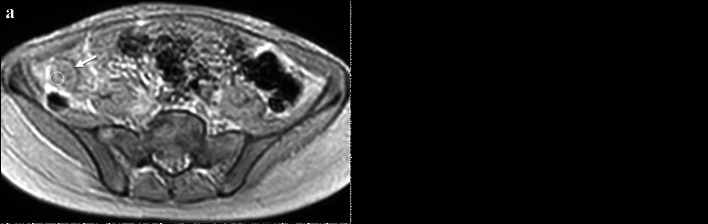
A 12-year-old female with Crohn’s disease. **a** Axial magnetization transfer (MT) MR image without an off-resonance radiofrequency (RF) pulse shows that the ileal wall is hyperintense (*arrow*). **b** Axial MR image with an off-resonance RF pulse shows loss of signal intensity in the bowel wall (*arrow*), presumably due to the presence of macromolecules. Regions of interest (white circles) were placed in the bowel wall on both sequences to calculate the MT ratio (45% for this patient)

Prior studies in animal models and adults have shown an association between bowel wall fibrosis and magnetization transfer ratio (MTR) measurements [41–43]. It is hypothesized that macromolecules in the bowel wall related to fibrosis such as collagen and extracellular matrix proteins lose signal with the application of an off-resonance magnetization transfer radiofrequency saturation pulse (Fig. 7) [44]. Greater loss of bowel wall signal can also likely be due to abnormally increased smooth muscle [45]. An adult study by Li et al. showed that bowel wall MTR normalized to skeletal muscle strongly correlated with histologic fibrosis scores (*r* = 0.77) [42]. These authors identified significant differences seen between nonfibrotic and mildly, moderately, and severely fibrotic bowel walls (*P* < 0.001) [42]. MTR measurements had an area under the receiver operating characteristic curve of 0.92 for differentiating moderately and severely fibrotic bowel walls from nonfibrotic and mildly fibrotic bowel walls [42]. In a recent mixed pediatric and adult study of 50 patients with CD undergoing small bowel resection and 83 patients with nonsurgical CD, normalized MTR was determined to be an independent predictor of the need for surgery when adjusted for other MRI features and clinical severity (OR = 1.07; *P* = 0.007) [16].

## Assessment of intestinal motility

Bowel segments affected by patients with CD show altered peristalsis [46]. Abnormal motility may be due to inflammation and/or fibrosis and can change over time with disease progression or treatment response. Several studies evaluating bowel motility in patients with CD have been performed to date, mostly in adult populations [47–49]. Cine MRI is most often evaluated in a subjective manner in the clinic. Specifically, the “frozen bowel sign” is commonly used to identify segments of bowel affected by CD and can be helpful for further characterizing bowel segments that are equivocally abnormal on other non-cine sequences [46].

**Fig. 8:**
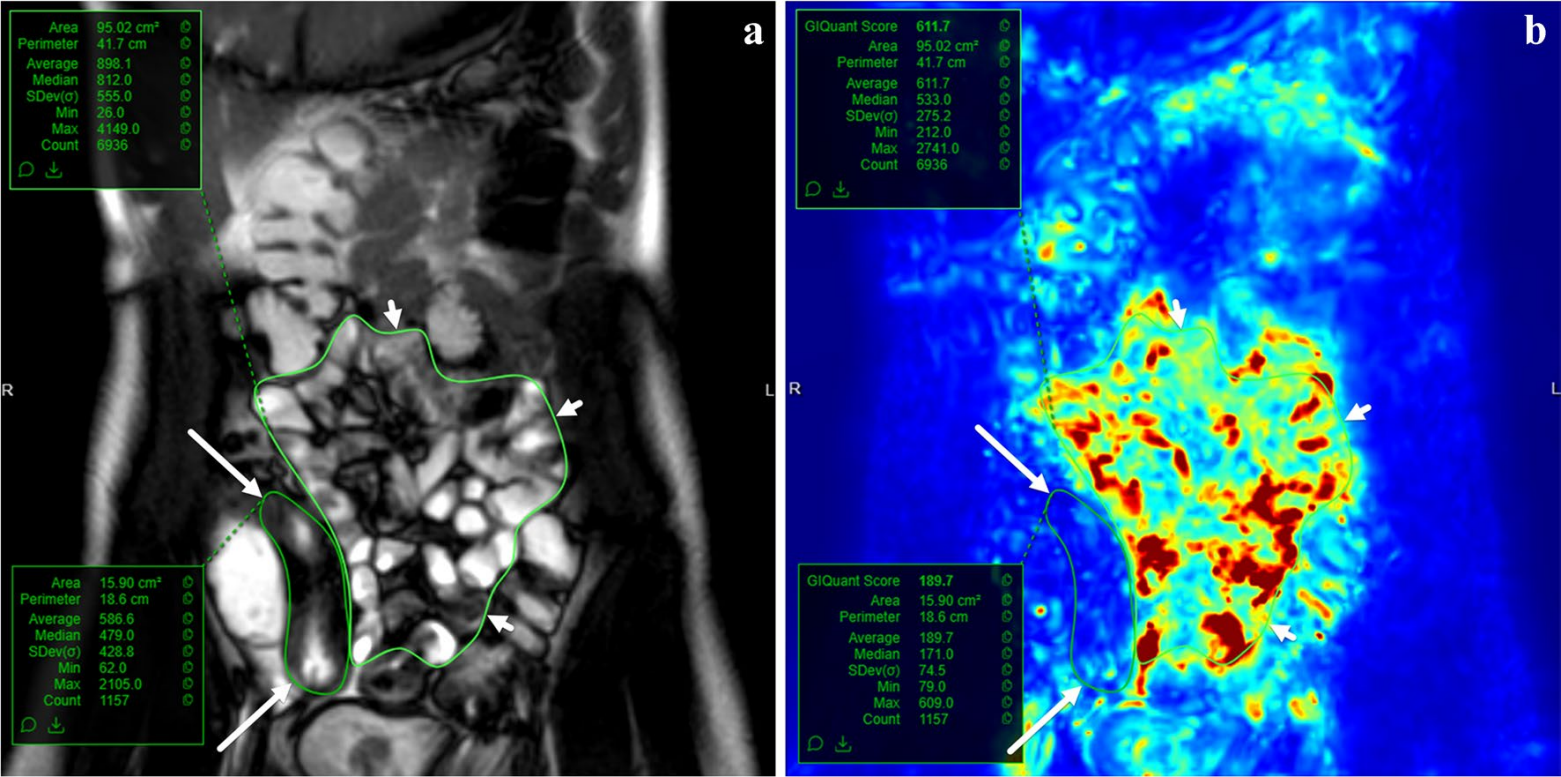
A 12-year-old female with Crohn’s disease. **a** Coronal two-dimensional balanced steady-state free precession MR image from a cine imaging sequence shows regions of interest encircling the terminal ileum (*long arrows*) and more proximal normal small bowel (*short arrows*). **b** Parametric map of intestinal motility shows that the terminal ileum (*long arrows*) has decreased peristalsis compared to more proximal bowel loops (*short arrows*) (*blue* is indicative of relatively little voxel motion compared to *red* and *yellow* which are indicative of relatively greater voxel motion)

Cine MRI of the bowel also can be evaluated using quantitative methods (Fig. 8). In a prospective study by Dillman et al., cine MRI was analyzed using voxel-based deformation field mapping in children with newly diagnosed ileal CD [50]. In that study, patients with CD were found to have decreased intestinal motility compared to healthy control subjects when normalized to motility measurements from fluid-filled, more proximal normal appearing bowel loops [50]. In addition, intestinal motility was shown to increase over time in response to medical therapy [50]. In their study, dynamic cine imaging was performed using a coronal 2D balanced steady-state free precession sequence at six to eight slice locations, including through the terminal ileum [50]. A US FDA-cleared tool (GIQuant; Motilent; London, UK) was used to process the images and measure intestinal motility. This tool had been previously validated and creates parametric motility maps based on deformation fields. Cococcioni et al. also quantitatively evaluated cine MRI in 25 children with CD or unclassified inflammatory bowel disease and showed that terminal ileal motility was lower in active disease and decreased with increasing histologic abnormalities [51].

While all of the above quantitative methods show promise for diagnosing and/or assessing treatment response, most techniques require further validation, particularly in children. In general, there is a current lack of pediatric-specific normative data as well as few studies assessing repeatability and reproducibility data. Additional work also is needed to understand the value of these methods (i.e., how they improve outcomes and/or lower healthcare costs), particularly when compared to standard of care diagnostic methods. Table 2 summarizes all the techniques listed in this review along with their advantages and disadvantages.

**Table 2:** Summary of different quantitative MRI techniques of patients with Crohn’s disease

Type	Technique	Reference	Use in pediatrics	Advantages	Disadvantages
Assessment of bowel wall inflammation	Magnetic Resonance Index of Activity (MaRIA)	[11]	[12]	• Calculated using routine clinical sequences • Strong correlation with endoscopy • Numerous studies validating in adults	• Requires intravenous contrast material • Cumbersome to use in clinical setting due to complex formula
	Simplified Magnetic Resonance Index of Activity (sMaRIA)	[14]	[15]	• Can be performed with routine clinical noncontrast sequences • Correlates with both original MaRIA and endoscopy • Does not require intravenous contrast material • Easy to use in the clinical setting • Substantial inter-reader agreement • Changes in response to medical therapy	• General lack of pediatric validation studies
	Pediatric Inflammatory Crohn’s Magnetic Resonance Enterography Index (PICMI)	[18]	[18]	• Developed in pediatric CD patients • Correlates with radiologist global assessment of inflammation and endoscopy • High inter-observer agreement • Changes in response to medical therapy	• Requires additional pediatric validation studies
	T1 relaxation mapping	[21]	[22]	• Correlates with clinical inflammatory markers, including C-reactive protein and the weighted Pediatric Crohn’s Disease Activity Index • Changes in response to medical therapy	• General lack of pediatric validation studies • Lack of repeatability/reproducibility data in children • Measurements are challenging in the absence of moderate-severe wall thickening
	Clermont score	[28]	[12]	• Correlates with both endoscopy and original MaRIA • Calculated using routine clinical sequences • Does not require intravenous contrast material	• General lack of pediatric validation studies • Cumbersome to use in clinical setting due to complex formula
	Diffusion-weighted imaging (DWI)	[23, 24]	[25, 26]	• Sequence commonly included in routine clinical pediatric MRE protocols • Numerous studies validating in children, including published ADC cut-off values for bowel wall active inflammation • Does not require intravenous contrast material • Changes in response to medical therapy	• Lack of repeatability/reproducibility data in children • Image quality may be suboptimal due to artifacts (e.g., motion, susceptibility) • Measurements are challenging in the absence of moderate-severe wall thickening
	Intra-voxel incoherent motion (IVIM)	[30]	[31]	• Sequence commonly included in routine clinical pediatric MRE protocols (requires additional scan time and *b*-values, however) • Provides intestinal perfusion metrics without intravenous contrast material	• Requires advanced post-processing • General lack of pediatric validation studies • Lack of repeatability/reproducibility data in children • Image quality may be suboptimal due to artifacts (e.g., motion, susceptibility) • Measurements are challenging in the absence of moderate-severe wall thickening
	Measurement of mesenteric blood flow	[36]	[35]	• Provides intestinal perfusion metrics, including blood flow (ml/min) • Can be measured in SMA or SMV using a standardized anatomic location • Does not require intravenous contrast material • Changes in response to medical therapy	• Requires pulse sequence that is not routinely acquired in clinical practice • Requires advanced post-processing • General lack of pediatric validation studies • Lack of repeatability/reproducibility data in children
Assessment of bowel wall fibrosis	Magnetization transfer ratio (MTR)	[41–43]	[16]	• Measurements shown to correlate with bowel wall fibrosis in animals and humans in multiple studies • May predict need for surgery • Does not require intravenous contrast material	• Requires pulse sequence that is not routinely acquired in clinical practice • Requires advanced post-processing • General lack of pediatric validation studies • Lack of repeatability/reproducibility data in children • Measurements are challenging in the absence of moderate-severe wall thickening
Assessment of intestinal motility	Cine MRI	[47–49]	[50, 51]	• Sequence commonly included in routine clinical pediatric MRE protocols • Numerous studies validating in children and adults • Does not require intravenous contrast material	• Requires advanced post-processing (single US FDA-approved tool to date)

*ADC*, apparent diffusion coefficient; *FDA*, Food and Drug Administration; *MRE*, magnetic resonance enterography; *SMA*, superior mesenteric artery; *SMV*, superior mesenteric vein

## Conclusion

In conclusion, numerous MRI techniques can be used to objectively assess intestinal inflammation, bowel wall damage, and motility in children with small bowel CD. Several of these quantitative methods are based on sequences that are routinely acquired in the clinic and that are presently evaluated in a qualitative manner. At present, there is a need to better understand the appropriate use of such quantitative assessments, their multiparametric performance, and how they impact meaningful patient outcomes in order to increase their use in the clinic.

## Data Availability

Our manuscript has no associated data since it is a review article.
